# Intense Body Contact Increases Homosexual Pair Bond Stability in Female Japanese Macaques (*Macaca fuscata*)

**DOI:** 10.1007/s10508-023-02781-6

**Published:** 2024-01-12

**Authors:** Pia Marlena Böhm, Lena Sophie Pflüger, Katharina Elisabeth Pink, Michael Alan Huffman, Bernard Wallner

**Affiliations:** 1 Department of Behavioral and Cognitive Biology, University of Vienna, Djerassiplatz 1, 1030, Vienna, Austria; 2 Austrian Research Center for Primatology, Ossiach, Austria; 3 Department of Evolutionary Anthropology, University of Vienna, Vienna, Austria; 4 Family and Population Studies, KU Leuven, Louvain, Belgium; 5 Wildlife Research Center, Kyoto University, Kyoto, Japan

**Keywords:** Female homosexuality, Japanese macaques, Pair bond, Consort, Huddling, Body contact

## Abstract

The Japanese macaque (*Macaca fuscata*) has become a key species for studying homosexual behavior over recent decades. With the non-conceptive nature, their same-sex consortships illustrate that individual partner preferences can exist beyond direct reproductive benefits or apparent sociosexual strategies. An open question is whether the behavior shared between partners in consortship directly affects their choice to remain with a partner. With this study, we examined behavioral aspects underlying consortship temporal patterns in these promiscuous and bisexual primates. While these patterns could be relevant in both homo- and heterosexual consortships, our study primarily focused on female-female pairs. We hypothesized that the stability of consortships (duration and occurrence) is influenced by a pair’s sexual behavior, mutual sexual stimulation, and close affiliative inter-mount behaviors involving high-intensity body contact. A semi-free population of Japanese macaques was observed over one mating season. In total, 40 h of focal data on female-female consortship behaviors were analyzed. Forty-six percent of all sexually mature females engaged in homosexual interactions. Our behavioral analyses of female-female pairs found that close body contact, rather than grooming or sexual interactions, was correlated with the stability of homosexual consortships. The greater the amount of huddling and embracing a pair engaged in, the more likely they were to stay together and reunite again. However, the frequency of mounting, rubbing or thrusting had no discernable effect on consortship stability. The results of this study thus add important knowledge to partner qualities in promiscuous primates as well as to inter-group differences of homosexual behavior in Japanese macaques.

**Supplementary Information:**

The online version contains supplementary material available at 10.1007/s10508-023-02781-6.

## Introduction

In most primate species females engage with multiple partners over the course of their reproductive stage in life. In seasonal breeding macaques, sexual activity is limited to the mating season, during which they form exclusive short-term relationships called consortships (consorts: Manson, 1997). The number of partners and the consort duration varies both between macaque species (Dixson, 2012) and individuals of the same species. Some females change their partners multiple times a day, while others stay with a preferred partner for multiple days or even reunite with the same partner more than once during a mating season (Huffman, 1991a; Takahata et al., 2002).

The most prevalent explanation to favor a partner over others is the factor of male dominance (Smuts, 1987). In macaques however, associations between dominance and reproductive success seem less stable than in other primate species (Paul, 2004). High-ranking males do not necessarily sire more offspring and might even be rejected by females more frequently than lower ranking males (Huffman, 1987, 1992; Takahata et al., 1999). Macaque females often prefer novel partners and rarely have more than one offspring from the same male (Inoue et al., 1991; Soltis, 2004). Individual preferences for selected consort partners have been observed in macaques including rhesus macaques (Manson, 1992) and Japanese macaques (Huffman, 1991a; Takahata, 1982a). Even more, some of those pairs kept recurring over multiple seasons and their mating consorts turned in some cases to stable, but non-sexual male–female relationships (Huffman, 1991a; Takahata, 1982a).

Thus, preferences for certain individuals not only exist, but can be maintained for years, beyond the initial sexual context. Partner preferences can even encompass non-conceptive partners. Rhesus macaque as well as chimpanzee males have been reported to favor non-fertile females over available fertile alternative partners (captive rhesus macaques: Herbert, 1970; captive chimpanzees: Allen, 1981, as cited in Smuts, 1987). In the end, the most evident partner preferences without reproductive benefit are preferences for same-sex partners. Same-sex sexual behavior is not an anomaly, it is wide-spread across many animal taxa (Monk et al., 2019). Females sexually interacting with other females, despite options for male partners, have been observed in many primate species such as chimpanzees (*Pan paniscus*), gorillas (*Gorilla gorilla*), rhesus macaques (*Macaca mulatta*), crested macaques (*Macaca nigra),* hanuman langurs (*Presbytis entellus*), and most famously, Japanese macaques (*Macaca fuscata*) (Vasey, 1995).

In Japanese macaques, homosexual behavior has been intensely studied over the last 50 years. This research could rule out many sociosexual alternative explanations for female-female choice, such as a lack of male partners (Leca et al., 2015a), forming alliances (Vasey, 1996), attracting male attention (Vasey, 1995), dominance displays (Huffman, 1987; Vasey et al., 1998), reconciliation (Vasey, 2004) or gaining allo-parental care (Vasey, 1998a). Yet, all studies have led to a common conclusion that female-female consorts are sexual in nature and evoke sexual pleasure while being non-conceptional. Their behavioral patterns, both in the mounting and inter-mount phase, are also comparable to heterosexual consorts. Sexually active females of all ages engage in homosexual consorts, even if they have access to alternative willing male partners (Vasey, 1998a; Vasey et al., 2014), and have preferred female partners (Leca et al., 2015a). Japanese macaque females actively mount and solicit each other reciprocally (Vasey et al., 1998), and show a diverse spectrum of mounting positions, leading to multifaceted ways of mutual stimulation (Vasey & Duckworth, 2006; Vasey et al., 2006).

With their non-conceptive nature, same-sex consorts illustrate that individual partner preferences can exist independent of direct benefits for reproduction and offspring fitness or sociosexual strategies. Instead, could these preferences be driven by factors directly shared between partners while engaging in consort relationships? Immediate effects of behaviors shared between partners remain understudied in promiscuous primate species in homo- and heterosexual consorts. One possibility is that females prefer to stay with partners that meet their sexual needs. In rats, sexual mounting is known as a rewarding behavior that activates the dopaminergic system (Melis & Argiolas, 1995). Female sexual stimulation is linked to oxytocin secretion, a hormone associated with the facilitation of pair bond formation (Carter, 1998). For example, GG-rubbing between female bonobos, involves direct genital stimulation, and has been shown to lead to an increase in oxytocin release (Moscovice et al., 2019). Yet, no studies have thus far looked at whether the frequency of sexual mounting could be connected to consort duration. In between mounting sequences, consort partners also engage in affiliative behaviors which are likely to influence the maintenance of their interactions. Behaviors characteristically shared during inter-mount phases are mutual following, social support, contact sitting, grooming and huddling (Vasey, 1998b; Vasey et al., 2008; Wolfe, 1984). Most of these behaviors imply body contact between two individuals. Body contact has been shown to reduce stress and increase overall well-being, while also reinforcing social ties (Hertenstein et al., 2007; Morrison, 2016). In primates, affiliative touch and body contact is considered a key element in the formation and maintenance of social relationships (Dunbar, 2010; Jablonski, 2021).

Physical touch facilitates emotional connection and promotes intimacy, trust, and cooperation within partnered relationships in humans and other primates (Field, 2014; Hertenstein et al., 2007). Grooming, hugging, and other tactile interactions also contribute to the release of oxytocin (Gothard & Fuglevand, 2022). A study investigating oxytocin’s effect in cotton-top tamarins, a pair-bonded monogamous species, found that a model including both huddling and grooming, but not sexual interaction, explained most of the variance in female oxytocin levels (Snowdon et al., 2010).

Whether shared body contact is a driving factor to strengthen potential consort bonds between partners has not yet been studied in Japanese macaques or any other promiscuous primate species. With this study, we aim to gain first insights into behavioral aspects underlying consortship patterns in a promiscuous, bisexual primate species, the Japanese macaque. While these patterns could be relevant in both homo- and heterosexual consorts, we have chosen to focus our study on female-female consorts first. By doing so, we aim to minimize the potential confounding factors such as social rank, age, and sexual coercion, which could influence female mate choice, but seem to be less important in female-female consorts (Vasey, 1996; Vasey et al., 1998). This allows us to isolate and explore the role of shared behaviors, particularly those involving body contact, in promoting the duration and recurrence of consorts. Given the mutuality of same-sex consort interactions, the female Japanese macaque represents a good model to discover affiliative interactions shared between partners in a consort and how this affects consort duration and recurrence.

We hypothesized that the stability of consorts between female Japanese macaques, as indicated by longer durations and repeated occurrences, is positively influenced by three key factors: (1) high frequencies of sexual behavior, (2) mutual sexual stimulation during consorts, and (3) close affiliative inter-mount behaviors involving high-intensity body contact. We predict that these behaviors play a vital role in strengthening consort bonds between partners. To test this, we investigated a semi-free ranging group of Japanese macaques (Affenberg Landskron, Austria) over the course of an entire mating season. We quantified hetero- and homosexual consorts, and qualified homosexual consorts by analyzing all behaviors that occurred between consort partners.

## Method

### Subjects

Behavioral observations were conducted on a semi-free ranging group of Japanese macaques (*Macaca fuscata*), living in a 40,000m^2^ naturally forested outdoor enclosure at the Affenberg Landskron in Carinthia, Austria. The population originated from the free-ranging Minoo H group located outside of Minoo city, Osaka Prefecture, Japan, and was relocated to Austria in 1996. For further information on population demographics and the facility, see Pflüger et al. (2021). During our study the group consisted of 165 individuals. From this, 80 were sexually matured females (≥ 3.5 years) and 49 sexually matured males (≥ 4.5 years) following Nakamichi and Yamada (2010). Data collected from females who died during the observation period (n = 4 females) were excluded, resulting in data from 76 females who entered our analyses. The resulting sex ratio of sexually matured males to sexually matured females was 1:1.55 (0.64); a sex ratio comparable to that of wild populations, which average 0.65 (Fooden & Aimi, 2005). To control group size, but avoid interfering with the females’ natural hormonal cycle, sterilization (tubal ligation) is routinely performed at the facility by veterinary staff after a female gives birth to at least one offspring. As a consequence, only 24 females were reproductively intact at the time of the present study. The females in the study group could be divided into seventeen matrilines of which eleven were represented by ≥ 5 individuals. All group members were individually identifiable via facial and body features and the ages of all individuals born in the group after relocation were known (Pflüger et al., 2021).

### Procedure

Data collection was conducted by a single observer (PMB) on an almost daily basis from September 26, 2019 to February 29, 2020, resulting in 126 observation days. There was no period longer than three consecutive days at a time when observations were not carried, with the exception of a twelve-day break over the Christmas holiday, due to Affenberg park management policy.

#### Quantification of Consorts

A consort was defined as an interaction between two individuals during which at least one mount was observed (see ethogram Table S1). As females also sexually mount each other, for our purposes a mount does not need to include intromission, thrusting or ejaculation.

A consort was regarded as one ongoing bout as long as a pair was observed repeatedly with no more than a three consecutive day gap in between observations. The duration of each consort bout was measured in days. We chose the three-day interval based on our data. The mean duration between two observations of the same pair was 3.8 ± 9.7 days. Therefore, by definition, the resumption of consort activity between a pair after four or more days was counted as a new consort between them (see *Consort Recurrence Rate*). Each potential consort pair sighted was only counted once per day, independent of the number of actual sightings made. If a pair was seen sitting in a ventro-dorsal contact position (for definition see Table S1), even without observing them mount on a day preceding or following a consort day with mounting, the pair was counted as still being in the same consort.

To quantify consort activity, hetero- and homosexual consorts of all individuals visible in a group were recorded using behavioral sampling (Martin & Bateson, 2007), whereby the whole group was observed throughout the day between 9 am and 5 pm, recording the presence of potential consort pairs. To avoid missing consorts that could be happening in less visible areas of the enclosure, the peripheral areas were additionally visited at least twice a day, at the start of the day and before leaving the enclosure (for a map of the enclosure see Pflüger et al., 2021). If a potential consort pair was found, the partners and their behaviors were recorded. We observed such pairs for 3 min, based on the reported average inter-mount interval of 1–2 min for Japanese macaques (Vasey et al., 2008). Female solicitation and inter-mount behavior were recorded and the direction of mounts categorized as male–female-mount (MFM), female-female-mount (FFM) or female-male-mount (FMM).

Both homo- and heterosexual consorts were quantified for the analysis of female consort activity, female access to male partners and the consort recurrence rate. Homosexual consort data was used for group level analysis of female pair stability, using the focal sampling data (see below).

#### Focal Sampling Protocol of Female Homosexual Pairs

Homosexual consort pairs were observed by focal sampling, whereby exact records of behaviors of the specified individuals were recorded in order to measure true frequencies and durations based on when behaviors started and ended (Martin & Bateson, 2007). Focal sampling started when the first mount between two females was observed (see ethogram Table S1). A given consort pair’s focal sampling session lasted 20 min (10 min per partner). The same pair was sampled once per day for each day the pair was spotted in consort.

Within each focal sampling session, the state of the consort was evaluated continuously. The active (actor) and passive (receiver) role of each member of the pair’s dyadic social interactions were recorded. If a pair separated from each other by more than 3 m during a focal sampling session, indicating the end of that particular interaction (see *Pair Consort Duration* below), the current focal individual of the pair was followed until the 10-min was completed. However, if the pair spatially separated during the first 10 min, and had not reunited by the end of that period, the same focal individual was followed for another 10 min to complete the 20-min focal sampling session. This was done to guarantee equal observation time for each pair, and detection of possible pair reunions within the full 20-min focal sampling session. A session was paused in cases when the focal individual moved out of sight. If the individual was found again within three hours after the sessions original starting time, that focal observation was resumed. A three hour limit was chosen to help guarantee that the original time of day conditions (light, food availability, hormonal state) remained comparable. If more than three hours passed, and the pair was not found again in consort, we discarded the focal sample data and started over if the pair were later relocated that day. Focal sampling sessions lasting less than 15 min (< 75% of 20-min focals) or those that did not meet all other criteria (n = 17), were also discarded and not used in the analysis.

The behavioral ethogram used for focal sampling observations is provided in Table S1.

#### Female Reproductive State

For further elucidation of reproductive age during the study period, females were categorized as adolescent (N = 10, 3.5 to 4.5 years) or mature adults (N = 66, > 5 years), corresponding to the age a female first showed signs of estrous or the proceeding years thereafter, respectively (following Nakamichi & Yamada, 2010). Since age alone is not predictive of a female’s hormonal and reproductive state (Wallner et al., 2011; Yoshida et al., 2001), we further categorized reproductive state into four categories: (1) nulliparous, (2) parous, (3) lactating (females with infants), and (4) menopausal-like females (≥ 26 years of age; Hamada & Yamamoto, 2010). All sterilized females under the age of 26 were categorized as adult and parous, since their cycles were not affected by tubal litigation, and they had given birth before.

### Measures

#### Variables Recorded During Behavioral Sampling

##### Female Consort Activity

Female consort activity was the total sum of consorts a female had throughout the observation period (as either homo- and heterosexual interactions), irrespective of the partners and the duration. However, if females had more than one consort partner in a day, each partner was counted separately for the purpose of quantifying other factors listed below.

##### Female Access to Male Partners

To analyze whether female homosexual activity was related to limited access to male partners, we assessed the number of different male consort partners per female. The reproductive outcome of all sexually mature, non-sterilized, females (n = 21) in the following birth season was compared to their homosexual activity throughout the previous mating season. In this way, we could assess the possible impact of homosexual consorts on a female’s reproductive success.

##### Consort Recurrence Rate [RR]

The RR of a pair (see PCD below) of a pair was calculated as the number of times a pair was observed in independent consorts together (for definition of a consort bout see above).

##### Total Days in Consort

The total days in consort (days observed) were calculated as the sum of individual days a pair was spotted in consort, irrespective of the recurrence rate and the focal observation data. The preceding and following days when ventro-dorsal contact sitting was observed were also included in calculating total consort duration.

#### Variables Recorded via Focal Sampling of Homosexual Consorts

##### Pair Consort Duration [PCD]

The PCD was calculated as the cumulative duration (in seconds) of consort behavior displayed by a pair across all focal sampling sessions of the pair.

Consort behavior included all instances of mounting, affiliative behaviours with body contact (embracing, contact sitting, huddling, grooming), cofeeding, traveling together or mutual following and a 3 m spatial proximity (3 m represent the measure of individual tolerance in Japanese macaques, Wada & Ogawa, 2009). PCD did not include times when partners stopped interacting and left their 3 m proximity radius. The resulting sum (in seconds) of consort behavior displayed between partners entered our statical analyses as PCD value. For PCD values in relation to total observation time of each pair please see Table S2.

##### Mounting [M]

The frequency of mounting was recorded across all focal sampling sessions of a pair, with a modifier for the occurrence of pelvic movement. For analysis, all mounting postures were combined into one variable to compare the mounting frequency, including both active and passive behaviors; Table S1.

##### Pelvic Movement [PM]

For every occurrence of mounting, the concurrence of pelvic movements (rubbing, grinding or thrusting) observed between a pair were recorded as a 0 or 1. This frequency of pelvic movement was corrected for with the frequency of all mounting as follows:

$${\text{PM}}= {\text{sum~pelvic~movements/sum~mounts}}$$

##### Mounting Reciprocity [MR]

Mounting reciprocity was calculated as the difference (in percentages) of active mounting frequencies between partners of a pair, subtracted from 100 as follows:

$$\mathrm{MR }= 100-({\mathrm{\% Active Mounting}}_{{\text{A}}} -{\mathrm{\% Active Mounting}}_{{\text{B}}} )$$

For example, if both partners actively and equally mounted the other, their mounting reciprocity value would be 100 (100 – (50%-50%)). If only one partner actively mounted within a consort, their pair mounting reciprocity would be 0 (100 – (100%-0%)). All mounting postures were considered (see Table S1).

##### Intensive Body Contact [IBC]

**Fig. 1:**
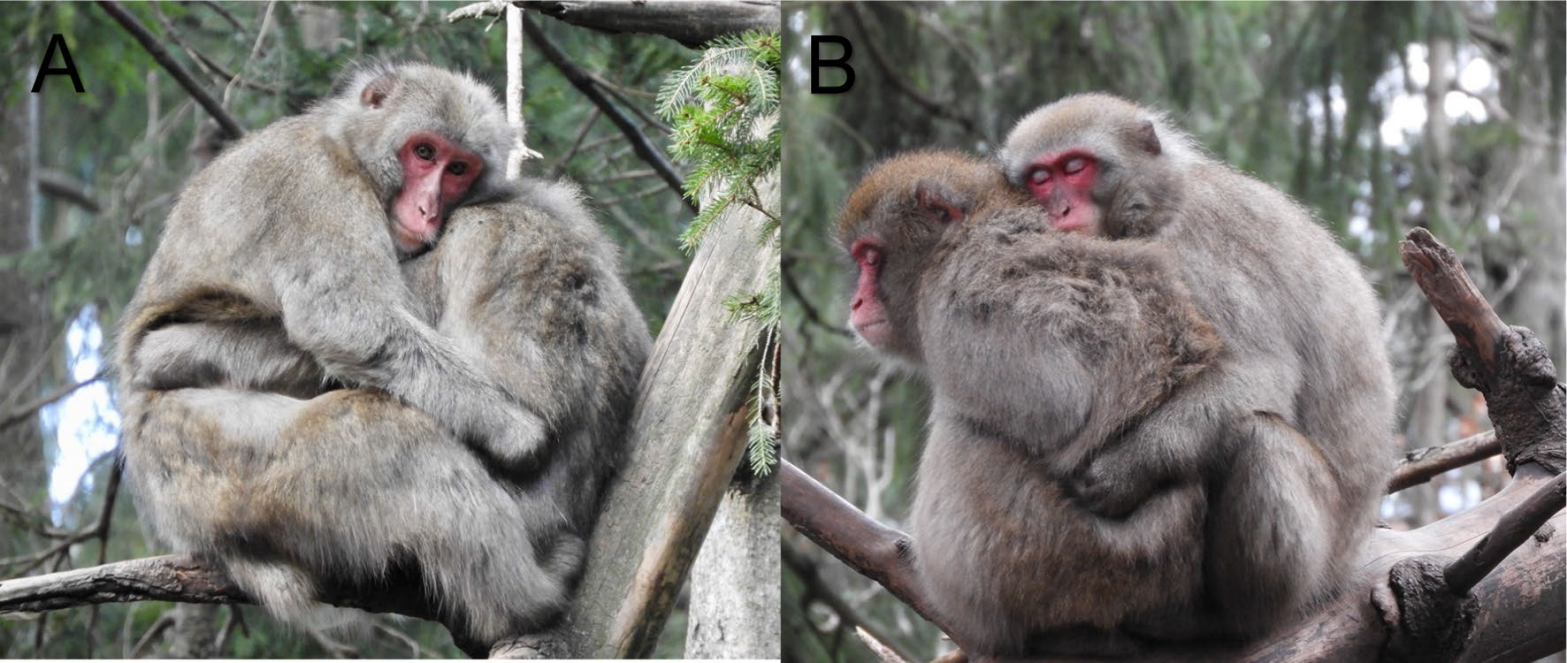
Examples of female-female huddling positions during a homosexual consort observed in the present study group (Affenberg Landskron, photos by PMB). (**a**) ventral and ventral huddling. (**b**) ventral and dorsal huddling. Definitions according to Ogawa and Wada (2011)

IBC was measured as the duration (in seconds) of any form of close, full surface body contact, including embracing, and huddling a pair shared across all focal sampling sessions (see Fig. 1). Contact sitting and grooming were treated separately and did not enter the IBC variable for the following reasons: (1) grooming is a directed behavior, in which body contact is limited to a limited body surface touched by the hands of the groomer, (2) contact sitting is defined as sitting next to another with continuous contact of any body part(s) in any position (e.g., back-to-back). This variable can be recorded in combination with other behaviors performed by the individual, such as feeding, and can be a pre-stage to mounting. In contrast, huddling and embracing are full surface contact positions with no movement. This leads to continuous body contact with both individuals actively participating in its maintenance.

##### Grooming [GR]

Grooming was measured as the duration (in seconds) of all grooming interactions between consort partners in both directions across all focal sampling sessions of the pair.

#### Consort Behavior and Pair Consort Stability

To assess the influence of the variables (M, PM, MR, IBC, GR) on the stability of a consort, we used pair consort duration (PCD) from the focal sampling data and the recurrence rate (RR) and the total days in consort (days observed) from the behavioral scan sampling data as our variables of interest. Each pair was assigned a unique ID. Some females were observed as partners in more than one pair, leading to 26 pairs that entered the analysis. Since pairs with more observation minutes (due to higher number of days observed in consort) have a greater probability of sharing a higher frequency/duration of behavior, the frequency of mounting (M) and the duration of intense body contact (IBC) and grooming (GR) were calculated as a proportion of their total focal observation time by dividing by the seconds of focal duration (TOD). The variable PM was corrected for by dividing PM by the total frequency of M (see above). This data from the 26 pairs observed can be found in Table S2.

### Data Analysis

All statistical analyses were performed in R (R Core Team, 2020), using the packages “Hmisc” (Harrell & Dupont, 2020) and “sjstats” (Lüdecke, 2020). For figures we used the packages “ggplot2” (Wickham, 2016) and “PerformanceAnalytics” (Peterson & Carl, 2020). We refrained from modelling our data due to the overall small sample size and instead focused on a more descriptive analysis. We used a Kruskal Wallis Test for group comparisons and a Mann–Whitney-U-test for pair comparisons. To relate PCD, the days observed (in consort), and RR to the described behaviors (M, IBC, PM, MR, GR), we created a correlation matrix with Spearman correlations using a Holm-Bonferroni correction.

## Results

### Female Consort Activity at the Group Level

Out of the 76 sexually mature females in the group, 71 were observed in consort. Of these, 69 formed heterosexual consorts with 1 to 14 different partners, resulting in 344 different female-male pairs. Thirty-five females were observed in homosexual consorts with 1 to 4 different partners, for a total of 31 different female-female pairs (Table 1).

**Table 1:** Female consort activity on the group level

		Overall (n = 71)	Homosexual (n = 35)	Heterosexual (n = 69)
consorts	Mean ± SD	8.7 ± 5.4	2.5 ± 1.9	7.7 ± 5.2
	range	1–25	1–8	1–23
partners	Mean ± SD	5.7 ± 3.4	1.8 ± 1.0	5.0 ± 3.2
	range	1–16	1–4	1–14

Females who engaged in homosexual consorts belonged to ten of the 11 functioning matrilines. Eight of the 31 homosexual pairs were observed in consort more than once during the mating season. These homosexual pairs had a mean consort recurrence rate of 1.75 bouts (SD ± 1.04 bouts, range: 1–4). No females were observed to have multiple homosexual consorts going on concurrently, though one female was recorded to switch from one homosexual consort partner to the next in the same observation day. Two cases were observed where females from the same matriline were in a homosexual consort, but neither of the pairs were related to each other within the 3rd degree of consanguinity.

**Fig. 2:**
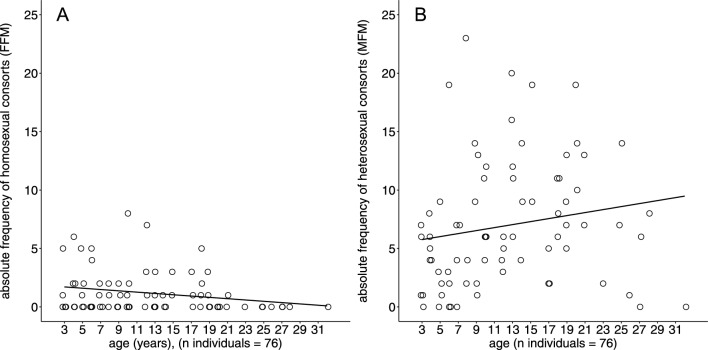
Scatterplot of the absolute frequencies of homosexual (**a**) and heterosexual (**b**) consort activity of all females (n = 76), categorized by age

A high level of inter-individual variation was found with regards to the overall consort activity among females of different ages and matrilines (Fig. 2, see also Table S3 for more details on the individual’s consort activity, age and matriline).

From the 26 homosexual pairs sampled, 112 consort observations of 20-min duration each were recorded (mean 7.8 observations ± SD 8.0 observations per pair with mean 87.2 min SD ± 86.0 min, range: 20–295 min). In 89% (n = 178) of these consorts, both partners were sampled for 10 min each. In another 11% (n = 22) only one of the partners were sampled for the full 20 min, and remaining five pairs had to be excluded because focal observation length was less than 15 min.

### Age and Homosexuality

The mean age of females participating in consorts (homo- and heterosexual) was 12.3 years (SD ± 6.7 years, range: 3–28). The mean age of homosexually active females was 10.7 (SD ± 5.4 years, range: 3–21). The mean age of females that were heterosexually active was 12.5 (SD ± 7.6 years, range: 3–28). Across the whole group (n = 76), age had no effect on homosexual consort activity (R_S_ = −0.2; *p* = 0.08) and only a slight positive effect on heterosexual consort activity (R_S_ = 0.3; *p* = 0.0075; Fig. 2).

**Fig. 3:**
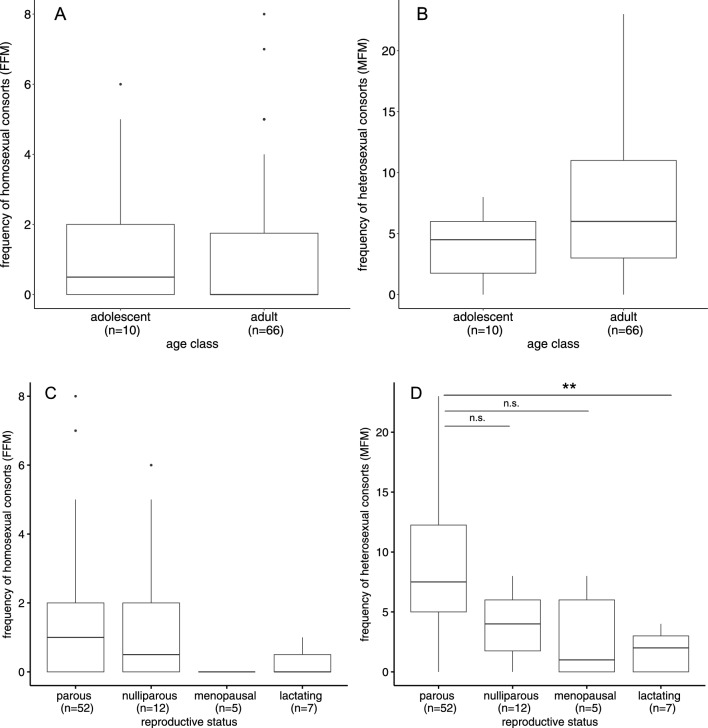
Consort activity in relation to age group and reproductive status. Boxplots of (**a**) the homosexual and (**b**) the heterosexual consort activity with age classes (adolescent [n = 10] and adult [n = 66]) and (**c**) the homosexual and (**d**) the heterosexual consort activity for the four reproductive status categories: nulliparous (n = 12), parous (n = 52), lactating (n = 7) post-menopausal (n = 5). Holm-Bonferroni corrected significance levels as stars (*p < .05, **p < .01, ***p < .001) were added to indicate group differences

There were no statistically significant differences between adolescent (age 3 and 4 years, n = 10) and adult females (age between 5 and 28 years, n = 66) with regards to their heterosexual activity (Z = −1.63, *p* = 0.1038), or homosexual activity (Z = 0.56, *p* = 0.58; Fig. 3).

### Reproductive Status and Homosexuality

Mean consort activity, both heterosexual and homosexual, was lower in lactating (n = 7) and menopausal females (n = 5) than nulliparous (n = 12) and parous females (n = 52) (Fig. 3). Overall, these groups were statistically different in their heterosexual activity (χ^2^ = 22.15; df = 3; *p* < 0.001), but not in in their homosexual activity (χ^2^ = 6.28, df = 3, *p* = 0.0987).

The differences in heterosexual activity observed between parous and lactating females were statistically significant (Z = -3.68, *p* = 0.0014), but all other pair-wise comparisons were not.

### Female Access to Male Partners

The high number of homosexual partners could not be explained by the number of available heterosexual partners (R_S_ = 0.0937, *p* = 0.421, n = 76), and the number of homosexual consorts was not explained by the number of heterosexual consorts (R_S_ = 0.040, *p* = 0.732, n = 76).

Reproductive success in the following birth season was not diminished for females who engaged in homosexual consorts. Of the 24 sexually mature, non-sterilized females in the group, 9 of the 11 who also had homosexual consorts gave birth in the following birth season. The two females that did not give birth were both adolescent (3.5 years) during the mating season and thus not likely to have become pregnant.

### Consort Behavior and Pair Consort Stability in Homosexual Pairs

Pairwise correlations of all variables of interest are presented in Table 2. The absolute number of days a pair was observed, correlated significantly both with their recurrence rate (RR) and the pair consort duration during focal observations (PCD). A higher amount of intense body contact (IBC) during consort focals was statistically significantly correlated with the recurrence rate (RR), the number of days observed in consort (days) and the focal pair consort duration (PCD) (Table 2).

**Table 2:** Pairwise correlation between the variables “days observed” (days), “recurrence rate” (RR), “pair consort duration” (PCD), “mounting reciprocity rate” (MR), “pelvic movement rate” (PM), “mounts/second of focal duration” (M), “intensive body contact*/*second of focal duration” (IBC) and "grooming/second of focal duration” (GR)

	**Days**	**RR**	**PCD**	**MR**	**PM**	**M**	**IBC**
**RR**	0.668**						
	*0.0046*						
**PCD**	0.890***	0.555					
	< *.0001*	*0.0721*					
**MR**	0.285	0.21	0.402				
	*1*	*1*	*0.7487*				
**PM**	0.365	0.032	0.415	0.418			
	*1*	*1*	*0.6689*	*0.6689*			
**M **	0.053	−0.058	0.326	0.656**	0.463		
	*1*	*1*	*1*	*0.0063*	*0.3603*		
**IBC**	0.738***	0.722***	0.697**	0.269	0.104	0.213	
	*0.0005*	*0.0008*	*0.0019*	*1*	*1*	*1*	
**GR**	0.016	−0.108	0.106	0.305	0.22	0.245	−0.042
	*1*	*1*	*1*	*1*	*1*	*1*	*1*

Holm*-*Bonferroni corrected significance levels as stars (*p < .05, **p < .01, ***p < .001) with *p*-values in the bottom rows in *cursive*

**Fig. 4:**
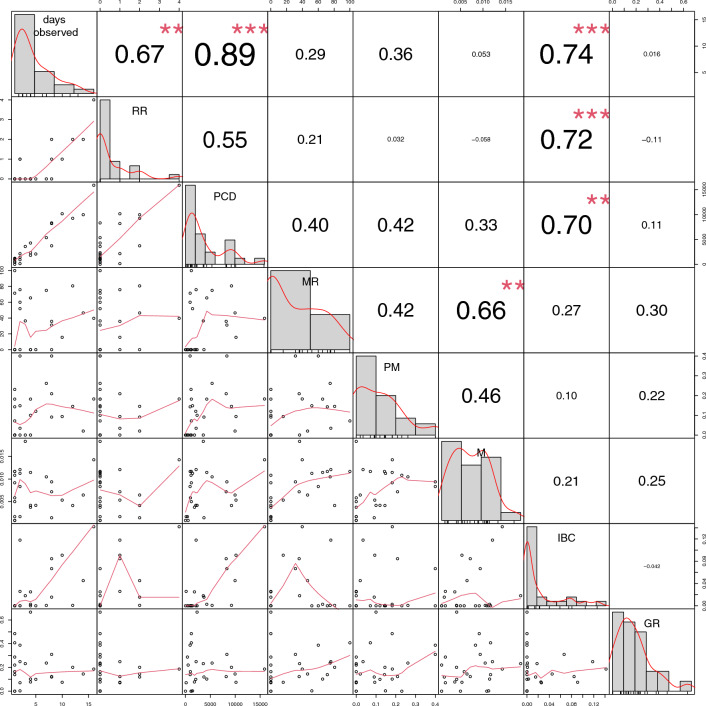
Correlation matrix plot of the variables “days observed”, “recurrence rate” (RR), “pair consort duration” (PCD), “mounting reciprocity rate” (MR), “pelvic movement mounts/mounting frequency” (PM), “mounts/second of focal duration” (M), “intensive body contact/second of focal duration” (IBC) and the “grooming/second of focal duration” (GR). On the diagonal, the distribution of each variable is displayed. Below the diagonal bivariate scatter plots with a fitted line are shown. The values of the correlation with their Holm-Bonferroni corrected significance levels as stars (*p < .05, **p < .01, ***p < .001) are shown above the diagonal

Relative mounting frequency (M) was not statistically significantly correlated with time spent in consort (RR, days, PCD). Neither was the reciprocity of mounting (MR), nor the rate of mounts involving pelvic movements (PM) or duration of grooming (GR) (Table 2; Fig. 4).

## Discussion

The aim of this study was to investigate the behavioral factors that drive partner preferences and consort stability in a promiscuous and bisexual macaque species. To pursue our research question, we studied the quality of female-female consortships in a semi-free ranging group of Japanese macaques (Affenberg, Austria). This provides the first detailed description of demographic factors underlying female consort activity with opposite and same sex partners. In contrast to other studies (Gunst et al., 2015), our study population showed no difference in frequency of homosexual consorts between adolescent and adult females, and displayed a comparably high prevalence of female homosexual behavior at the group level (Leca et al., 2014).

Our behavioral analyses of female-female consort pairs during an entire mating period revealed that close body contact, rather than grooming or sexual interactions, was correlated with the stability and duration of these homosexual consorts. Pair bonding and partner preference in primates is complex, being influenced by various interacting factors (Manson, 1992; Smuts, 1987; Soltis, 2004; Takahata et al., 1999). The dyadic behaviors shared between partners during a consort that affect its length had not been investigated before our study. Our hypothesis that the duration of a female-female consort is dependent on their sexual activity could not be supported. However, our results revealed that the consort length positively correlated with the amount of intense body contact shared between two female partners. That is, females who spent more time huddling and embracing each other stayed in consort longer and reunited more often than those who showed lower levels of these behaviors. These findings challenge the prevailing assumption that consort relationships are solely driven by sexual motivations and highlight the significance of close body contact in maintaining pair bonds.

The importance of close body-contact is consistent with studies on humans and other non-human primates that highlight the positive effects of affiliative touch and body contact in the context of pair bonding (Dunbar, 2010; Gothard & Fuglevand, 2022; Jablonski, 2021). For instance, a study on cotton-top tamarins found that huddling and grooming may be more important than sexual activity, as they significantly influence a female’s oxytocin level (Snowdon et al., 2010). Oxytocin, widely known as the “cuddling hormone,” has been the focus of numerous studies and is associated with both pair bonding and stress relief (Chen et al., 2020; Jakubiak & Feeney, 2019; Kreuder et al., 2017; Morrison, 2016; Portnova et al., 2020; Wallner et al., 2006).

In contrast to huddling and embracing, time spent social grooming did not correlate with the time females spent in consorts. In our analysis, the variable “*social grooming*”, was treated independently of intense body contact from the start. Social grooming, an intimate gesture performed between two individuals, is known to facilitate the maintenance of long-term social relationships based on mutual trust and reciprocity (Jablonski, 2021). While social grooming and huddling are both behaviors strongly associated with close social-bonds and kinship (Majolo et al., 2010; Ogawa & Wada, 2011), we believe that the mutual and extensive body contact shared in a pair-huddle or an embrace are of a different intensity than grooming. The quality and intimacy of social grooming depends on factors such as reciprocity, techniques and targeted body parts (Borries, 1992). In contrast, huddling and embracing involve mutual and tight body contact, often with both females’ faces in very close proximity (Ogawa & Wada, 2011, see Fig. 1), indicating high tolerance and affinity (Wada & Ogawa, 2009). The mechanical properties of deep pressure social touch in hugging and light pressure C-tactile (CT) touch of grooming likely activates different afferents (Field, 2019). It is therefore possible that grooming and cuddling, although both related to pair bonding, differ in their impact and underlying mechanisms. A further examination of the effects of different types of touch and body contact on pair stability and underlying physiological reactions is needed.

The question remains, whether any type of body contact in a sexual context can facilitate long-term relationships between individuals. Consortships are typically formed between individuals that do not already share an affiliative relationship outside of the mating season, and their relationship is limited to the few days spent in consort. However, in heterosexual male–female pairs, some consortships have been observed to not only have above average consort durations with selected partners, but recur over two or three years, leading to stable and long-term non-sexual associations, known as peculiar proximate relationships in the literature (Huffman & Takahata, 2012; Takahata, 1982a). These associations have been reported in studies of the Arashiyama (Huffman, 1991b; Takahata, 1982a, b) and Minoo populations (Perloe, 1992). It is possible that female-female homosexual consortships might also develop into similar stable year-round relationships if the partner preference between individuals is upheld for consecutive mating seasons. Longitudinal studies of these relationships are needed to investigate whether intense body contact during sexual interactions qualifies as a social bonding variable to develop into year-round non-kin related grooming and proximity relationships.

We acknowledge that our study was conducted only on female-female pairs during one single mating season in a semi-free ranging population of Japanese macaques. However, by focusing on female-female pairs, we minimized potential confounding factors such as social rank, age, and sexual coercion, which could influence female mate choice and seem to be less important in female-female consorts (Vasey, 1996; Vasey et al., 1998). By doing so, we were able to gain a first look into the role of social behaviors in short term consorts potentially influencing the duration and recurrence of consorts. It remains important to replicate our study in heterosexual pairs to investigate the importance of social touch in heterosexual consorts as well. An increased sample size is needed to be able to control for factors such as social rank, novelty of males and individual differences and preferences. Collecting and comparing data on hetero- and homosexual pairs from different Japanese macaque populations will help to increase our understanding of partner preferences and consort patterns in this species. This is of particular interest as not all Japanese macaque populations previously studied show homosexual behavior.

Comparing the prevalence of homosexual behavior between populations sheds light on potential inter-group differences and the influence of specific demographics on the expression of same-sex consorts (Leca et al., 2014; Vasey & Jiskoot, 2010). So far, this behavior has been associated with the specific haplotype A1 carried by populations located on the main island of Honshu (Vasey & Jiskoot, 2010). The Minoo population, from which the Affenberg group originates, belongs to this haplotype group. Female homosexuality was not only present but even more prevalent (46%) in the Affenberg group than free-ranging populations studied in Japan (i.e., Arashiyama-E 28.4%, Jigokudani-A1 0%, Minoo-F 11.3%, Minoo-L 10.6%; Leca et al., 2014).

This large number of female-female pairs might be explained by the novelty hypothesis, which suggests that females prefer partners they are unfamiliar with in terms of relations and prior experiences (Huffman, 1981). This hypothesis is supported by findings from semi-free-ranging groups, such as the Arashiyama-West group in Texas, which also exhibited a very high prevalence of female homosexual behavior (Wolfe, 1984).

Interestingly, our study found no relationship between the frequency of homosexual behavior and female access to male partners, indicating that homosexual consort activity is independent of a female’s heterosexual activity. This also holds true for adolescent females. In the present study group, females were likely to start reproducing after reaching the age of five (mean age at first birth = 4.92 years ± SD 0.87 years; Pflüger et al., 2021), and all females who engaged in homosexual interactions gave birth in the subsequent birth season, providing convincing evidence of female access to male partners.

Female homosexual behavior was widely distributed across different age groups and matrilines in the study population. There was no difference observed between adolescent and adult females, in contrast to previous studies (Leca et al., 2015b). The observed negative trend of age is likely to be influenced by the low incidence of homosexual behavior in females older than 18 years, which is expected to be due to reduced sexual activity in very old, likely menopausal-like and lactating females (Yoshida et al., 2001).

To conclude, our study provides first insights into the dynamics of body contact and its implications for pair bonding in promiscuous non-human primates. While our study adds to the knowledge of primate sexual and social relationships, it calls for future investigations into the underlying mechanisms and long-term consequences. We endorse future studies replicating our results in other Japanese macaque populations and even other promiscuous primate species. We suggest to use long-term observations to gain a more comprehensive understanding of consort dynamics and to investigate the potential influence of underlying hormone levels (Carter, 1998; Snowdon et al., 2010) and cycle stages (O’Neill, 2012) on long-term partner preferences.

### Supplementary Information

Below is the link to the electronic supplementary material.

Supplementary file1 (DOCX 33 kb)
