# Supplementary material for: Intense Body Contact Increases Homosexual Pair Bond Stability in Female Japanese Macaques (Macaca fuscata)
Source: Arch Sex Behav. 2024 Jan 12;53(5):1653–65. doi: 10.1007/s10508-023-02781-6 (PMC11106093; doi:10.1007/s10508-023-02781-6)
Supplement: Supplementary file 1 — Supplementary file1 (DOCX 33 kb) [file 10508_2023_2781_MOESM1_ESM.docx]

## Supplementary Material

| **Table S1** Ethogram |
| --- |
| **Aggressive consort interruption** Focal interrupts a consortship by aggressively trying to  displace one or both consort partners or make them split their proximity.  Possibly with body contact (hit/bite/grab) or without (chase/lunge/threat). |
| **Non-aggressive consort interruption:** Focal interrupts consort without showing aggressive  behaviour but displacing one or both consorting partners or make them split  their proximity. |
| **Allogrooming** Involvement in a multiple grooming interaction with at least two other individuals. |
| **Approach** Focal animal comes from beyond to within 1m perimeter (=proximity) of another individual, having focus on the approached individual. |
| **Attempted mount** Attempting a mount posture (described below) but failing to fully execute it (e.g. because the partner refused by not rising or because of an intervention or rejection). |
| **Body contact** Focal maintains contact with the body of another individual. |
| **Copulation/Consort Call** Focal vocalizes towards a (potential) sexual partner. Can be continuous  when pauses between copulation calls are shorter than 10 seconds |
| **Cry for support** Focal makes a high pitch vocalization accompanied by hectic looking and  searching for another individual during or after an aggressive encounter. |
| **Complain** loud whining and screaming, with a tensed or cowering body posture, without looking for support |
| **Co-feed** Focal is feeding/foraging with another feeding/foraging individual while being  within 1 meter from each other. |
| **Contact (Consort) sit** Focal animal is sitting in body contact with another individual face to back or back to face (ventral-dorsal orientation) |
| **Depart** Focal animal actively moves away and leaves the proximity radius of another individual. Departing requires the individuals to have been in proximity or other close interaction (grooming, contact sit) before and is a neutral exit from the previous state, with no aggression or dominance involved between the individuals. |
| **Embrace (hug)** One (or both) individual holding another in its arms |
| **Hold bottom** Focal individual is holding the rump of another individual while sitting in a consort sit |
| **Pull back** An individual reaches for their consort partner that is about to leave, pulling them back into proximity or a contact sit position. |
| **Refusal** Focal leaves or changes position while another individual either shows hold bottom, presents or solicits focal individual; focal is not showing aggression. |
| **Rejection** Focal aggresses an individual that has been trying to gain their attention (through presentation, solicitation or copulation calls). |
| **Ejaculation (/mating)** Male pauses after multiple thrusts and ejaculates. Afterwards semen should be visible on the female’s rump. |
| **Follow** Focal walks behind another individual in the same direction and stays within  a radius of 5 meters |
| **Groom give, groom receive – Grooming [GG, GR]** The receiver’s fur is touched by the actor with both hands, accompanied by periodic hand contact with the actor’s mouth |
| **Groom present** Laying down near another individual (within 1m), can lead to grooming |
| **Grunt** low pitched sound, used in different contexts e.g. threats, but also grooming |
| **Huddle** Individuals cuddling in closely, body contact maximized, head laying on the other individual |
| **Look back** Focal is turning their head around to look towards the consort partner while being mounted. |
| **Mount types** |
| ***Foot-clasp mount*** In which the mounter grasps between the mountee’s ankles and hips with his or her feet, and on the mountee’s back with his or her hands. |
| ***Miscellaneous mount*** Other, not classified, position, in which one monkey rubs their anogenital region against a body part of another monkey |
| ***Som*** *(****Sitting Mount)*** With the mounter sitting on the mountee’s back in a jockey-like position, while grasping the mountee’s upper back with his or her hands and the mountee’s lower back with his or her feet. |
| ***Standing Mount*** The mounter stands bipedally with his or her feet on the ground and his or her knees slightly bent, while grasping the mountee’s lower back with his or her hands |
| ***Reclined mount*** The mounter is lying ventrally on partners back, using his or her feet to grasp the mountee’s legs above the ankles and his or her hands to grasp fur on the partners upper back |
| ***Unspecified mount*** Any mount that was not recorded as a specific type but just as “mount” and therefore cannot be counted to a category but to the overall number of mounts |
| ***Ventral Mount*** Embracing the partner face to face while rubbing their perineum against the other’s anogenital region.  *sitting* - both females are sitting (F/F), rubbing against each other, pulling each other in to a tight embrace  *clinging* - one female stands, the other one is clinging to her from beneath, like an infant being ventral carried  *“classic”*- one female is lying on her back, the other one rubbing above her ventrally. Like a "missionary" position |
| **Muzzle Contact/kiss** Animal brings their own mouth region close to another individual’s and sniffs or kisses their partner (calmly) |
| **Present** Individual displays its rump and/or anogenital region to another by bowing forward and raising hindquarter. Includes all instances in which the female got up (or attempted to do so) and directed the rump towards her consort partner and/or bobbed the head slightly downwards. |
| **Proximity** (**Close spatial cohesion)** Focal animal is within 1m but further than body contact from another individual |
| **Reach back(Encouragement*)*** Focal is turning around to reach with one hand towards the consort partner, grabbing or touching them, while being mounted. |
| **Scream** High pitched, intense screaming |
| **Solicitation** Focal actively grabs or pulls the arm or other body parts of their consort partner to invite them for mounting. May be immediately followed by hindquarter presentation and looking at partner |
| **Travel together** Two individuals travel parallel to each other, distance between individuals is less than 3m. |

| **Table S2**.: Homosexual Pair Variables. Days of observation (days observed), Recurrence rate (RR), Pair consort duration (PCD), total observation duration (TOD), ratio pair consort to focal duration (RPCFD), Total mount frequency (M), Pelvic movement (PM), Pelvic Movement rate (PM), Mounting reciprocity rate as difference of active mounts in a pair (MR), Intense body contact (IBC), Grooming rate (GR); The variables M, IBC and GR were corrected for the total observation time (TOD) before entering our analyses. | | | | | | | | | | | | | | |
| --- | --- | --- | --- | --- | --- | --- | --- | --- | --- | --- | --- | --- | --- | --- |
| **PairID** | **days observed** | **RR** | **PCD**  **(s)** | **TOD**  **(s)** | **RPCFD(%)** | **M**  **(total)** | **PM**  **(total)** | **PM/M** | **MR** | **IBC**  **(s)** | **GR (s)** | **M/**  **TOD** | **IBC/**  **TOD** | **GR/**  **TOD** |
| AliceHeidi | 1 | 0 | 1200 | 1200 | 100.0 | 14 | 1 | 0.071429 | 100 | 0 | 489 | 0 | 0.011667 | 0.4075 |
| AmyGreta | 16 | 4 | 15886 | 17673 | 89.9 | 252 | 46 | 0.18254 | 39.68254 | 2508 | 3294 | 0.141911 | 0.014259 | 0.186386 |
| AugustineKate | 5 | 0 | 2086 | 6000 | 34.8 | 25 | 3 | 0.12 | 0 | 0 | 599 | 0 | 0.004167 | 0.099833 |
| AugustineMontana | 4 | 0 | 2350 | 2400 | 97.9 | 10 | 1 | 0.1 | 0 | 0 | 1599 | 0 | 0.004167 | 0.66625 |
| AugustineNora | 1 | 0 | 24 | 1200 | 2.0 | 1 | 0 | 0 | 0 | 0 | 0 | 0 | 0.000833 | 0 |
| BarbaraMadeleine | 1 | 0 | 1136 | 1200 | 94.7 | 13 | 3 | 0.230769 | 0 | 0 | 0 | 0 | 0.010833 | 0 |
| BertaElli | 1 | 0 | 1200 | 1200 | 100.0 | 11 | 2 | 0.181818 | 0 | 0 | 582 | 0 | 0.009167 | 0.485 |
| BumbumHeidi | 1 | 0 | 1012 | 1200 | 84.3 | 7 | 0 | 0 | 0 | 0 | 197 | 0 | 0.005833 | 0.164167 |
| CatwomanKrato | 8 | 0 | 8327 | 9600 | 86.7 | 90 | 36 | 0.4 | 31.11111 | 640 | 2973 | 0.066667 | 0.009375 | 0.309688 |
| CatwomanLilly | 2 | 0 | 1443 | 2400 | 60.1 | 27 | 4 | 0.148148 | 51.85185 | 0 | 6 | 0 | 0.01125 | 0.0025 |
| CatwomanMadeleine | 10 | 1 | 10160 | 12000 | 84.7 | 64 | 6 | 0.09375 | 15.625 | 1090 | 898 | 0.090833 | 0.005333 | 0.074833 |
| ClaudiaMadeleine | 1 | 0 | 1200 | 1200 | 100.0 | 6 | 0 | 0 | 0 | 0 | 0 | 0 | 0.005 | 0 |
| EvaGreta | 1 | 0 | 960 | 1200 | 80.0 | 14 | 2 | 0.142857 | 71.42857 | 0 | 167 | 0 | 0.011667 | 0.139167 |
| EvaMarlene | 1 | 0 | 555 | 1200 | 46.3 | 1 | 0 | 0 | 0 | 0 | 264 | 0 | 0.000833 | 0.22 |
| FannyIlvy | 8 | 1 | 8191 | 9336 | 87.7 | 67 | 14 | 0.208955 | 35.8209 | 789 | 1147 | 0.084512 | 0.007177 | 0.122858 |
| FlorentineFriderike | 12 | 2 | 9252 | 15000 | 61.7 | 62 | 9 | 0.145161 | 80.64516 | 12 | 3016 | 0.0008 | 0.004133 | 0.201067 |
| FlorentineIlvy | 2 | 1 | 157 | 1200 | 13.1 | 2 | 0 | 0 | 0 | 31 | 85 | 0.025833 | 0.001667 | 0.070833 |
| FlorentineNils | 8 | 2 | 3731 | 7739 | 48.2 | 11 | 1 | 0.090909 | 0 | 120 | 924 | 0.015506 | 0.001421 | 0.119395 |
| HermineKrato | 7 | 0 | 5365 | 8400 | 63.9 | 88 | 23 | 0.261364 | 75 | 6 | 1991 | 0.000714 | 0.010476 | 0.237024 |
| IlvyRudolfine | 14 | 2 | 9971 | 15339 | 65.0 | 99 | 2 | 0.020202 | 46.46465 | 693 | 2220 | 0.045179 | 0.006454 | 0.144729 |
| JanisLilly | 2 | 1 | 2156 | 2400 | 89.8 | 29 | 1 | 0.034483 | 75.86207 | 283 | 597 | 0.117917 | 0.012083 | 0.24875 |
| JuliaMarianne | 1 | 0 | 414 | 1200 | 34.5 | 2 | 0 | 0 | 0 | 0 | 345 | 0 | 0.001667 | 0.2875 |
| KateMelanie | 4 | 0 | 1822 | 4800 | 38.0 | 18 | 0 | 0 | 0 | 120 | 372 | 0.025 | 0.00375 | 0.0775 |
| KratoKurt | 3 | 0 | 3600 | 3600 | 100.0 | 66 | 0 | 0 | 36.36364 | 65 | 836 | 0.018056 | 0.018333 | 0.232222 |
| LillyMadeleine | 4 | 0 | 4293 | 4800 | 89.4 | 55 | 10 | 0.181818 | 65.45455 | 13 | 490 | 0.002708 | 0.011458 | 0.102083 |
| LillyZarah | 2 | 0 | 947 | 1200 | 78.9 | 10 | 4 | 0.4 | 60 | 0 | 465 | 0 | 0.008333 | 0.3875 |

| **Table S3** Consort activity and demographic factors of all sexually mature females (n = 76) | | | | | | | | | | | | |
| --- | --- | --- | --- | --- | --- | --- | --- | --- | --- | --- | --- | --- |
|  | consorts | | | partners | | |  |  |  |  |  |  |
| ID | all | MF | FF | all | MF | FF | age | age group | matriline | maturity | intact | Offspring 2020 |
| Kate | 25 | 23 | 2 | 11 | 9 | 2 | 8 | adult | 2 | parous | 0 | 0 |
| Krato | 23 | 19 | 4 | 16 | 12 | 4 | 6 | adult | 6 | parous | 0 | 0 |
| Hilde | 20 | 20 | 0 | 14 | 14 | 0 | 13 | adult | 8 | parous | 0 | 0 |
| Marianne | 20 | 19 | 1 | 12 | 11 | 1 | 15 | adult | 2 | parous | 0 | 0 |
| Laura | 19 | 19 | 0 | 12 | 12 | 0 | 20 | adult | 9 | parous | 0 | 0 |
| Beate | 16 | 16 | 0 | 8 | 8 | 0 | 13 | adult | 6 | parous | 0 | 0 |
| Berta | 15 | 14 | 1 | 12 | 11 | 1 | 14 | adult | 5 | parous | 0 | 0 |
| Fanny | 15 | 13 | 2 | 9 | 8 | 1 | 9 | adult | 13 | parous | 0 | 0 |
| Alice | 14 | 12 | 2 | 10 | 9 | 2 | 10 | adult | 8 | parous | 0 | 0 |
| Florentine | 14 | 6 | 8 | 7 | 4 | 3 | 10 | adult | 12 | parous | 0 | 0 |
| Kruemel | 14 | 14 | 0 | 11 | 11 | 0 | 25 | adult | 10 | parous | 0 | 0 |
| Luise | 14 | 14 | 0 | 4 | 4 | 0 | 20 | adult | 1 | parous | 0 | 0 |
| Nora | 14 | 13 | 1 | 10 | 9 | 1 | 21 | adult | 14 | parous | 0 | 0 |
| Simone | 14 | 14 | 0 | 6 | 6 | 0 | 9 | adult | 10 | parous | 0 | 0 |
| Ilvy | 13 | 6 | 7 | 8 | 5 | 3 | 12 | adult | 7 | parous | 0 | 0 |
| Josefine | 13 | 13 | 0 | 7 | 7 | 0 | 19 | adult | 5 | parous | 0 | 0 |
| Lilly | 13 | 8 | 5 | 9 | 5 | 4 | 18 | adult | 5 | parous | 0 | 0 |
| Melanie | 13 | 12 | 1 | 10 | 9 | 1 | 13 | adult | 6 | parous | 0 | 0 |
| Augustine | 12 | 9 | 3 | 7 | 4 | 3 | 15 | adult | 6 | parous | 0 | 0 |
| Iris | 12 | 11 | 1 | 9 | 8 | 1 | 18 | adult | 2 | parous | 0 | 0 |
| Caroline | 11 | 11 | 0 | 7 | 7 | 0 | 18 | adult | 10 | parous | 0 | 0 |
| Catwoman | 11 | 6 | 5 | 6 | 2 | 4 | 3 | adolescent | 10 | nulliparous | 1 | 0 |
| Janis | 11 | 9 | 2 | 4 | 3 | 1 | 5 | adult | 8 | parous | 1 | 1 |
| Olga | 11 | 11 | 0 | 10 | 10 | 0 | 13 | adult | 10 | parous | 0 | 0 |
| Romana | 11 | 11 | 0 | 2 | 2 | 0 | 10 | adult | 6 | parous | 0 | 0 |
| Betty | 10 | 10 | 0 | 6 | 6 | 0 | 20 | adult | 7 | parous | 0 | 0 |
| Greta | 10 | 4 | 6 | 4 | 2 | 2 | 4 | adolescent | 7 | nulliparous | 1 | 1 |
| Dolores | 9 | 9 | 0 | 8 | 8 | 0 | 14 | adult | 9 | parous | 0 | 0 |
| Eva | 9 | 7 | 2 | 8 | 6 | 2 | 7 | adult | 12 | parous | 0 | 0 |
| Friderike | 9 | 6 | 3 | 5 | 4 | 1 | 13 | adult | 7 | parous | 0 | 0 |
| Lena | 9 | 9 | 0 | 7 | 7 | 0 | 19 | adult | 4 | parous | 0 | 0 |
| Suzanna | 9 | 9 | 0 | 5 | 5 | 0 | 9 | adult | 7 | parous | 1 | 1 |
| Barbara | 8 | 7 | 1 | 8 | 7 | 1 | 19 | adult | 2 | parous | 0 | 0 |
| Grace | 8 | 8 | 0 | 5 | 5 | 0 | 4 | adolescent | 12 | nulliparous | 1 | 1 |
| Heidi | 8 | 5 | 3 | 7 | 5 | 3 | 12 | adult | 2 | parous | 0 | 0 |
| Kathi | 8 | 8 | 0 | 6 | 6 | 0 | 28 | adult | 15 | menopausal | 0 | 0 |
| Lisa | 8 | 6 | 2 | 6 | 4 | 2 | 18 | adult | 6 | parous | 0 | 0 |
| Marlene | 8 | 6 | 2 | 4 | 2 | 2 | 4 | adolescent | 7 | nulliparous | 1 | 1 |
| Rudolfine | 8 | 5 | 3 | 5 | 4 | 1 | 19 | adult | 12 | parous | 0 | 0 |
| Claudia | 7 | 6 | 1 | 6 | 5 | 1 | 10 | adult | 5 | parous | 0 | 0 |
| Pippi | 7 | 7 | 0 | 3 | 3 | 0 | 3 | adolescent | 3 | nulliparous | 1 | 0 |
| Tanja | 7 | 7 | 0 | 4 | 4 | 0 | 25 | adult | 17 | parous | 0 | 0 |
| Ulrike | 7 | 7 | 0 | 7 | 7 | 0 | 21 | adult | 3 | parous | 0 | 0 |
| Uschi | 7 | 7 | 0 | 3 | 3 | 0 | 7 | adult | 8 | parous | 0 | 0 |
| Zarah | 7 | 5 | 2 | 6 | 4 | 2 | 4 | adolescent | 8 | nulliparous | 1 | 1 |
| Amy | 6 | 1 | 5 | 2 | 1 | 1 | 5 | adult | 12 | parous | 1 | 1 |
| Bumbum | 6 | 5 | 1 | 3 | 2 | 1 | 17 | adult | 3 | parous | 0 | 0 |
| Chanel | 6 | 6 | 0 | 4 | 4 | 0 | 10 | adult | 7 | parous | 0 | 0 |
| Michaela | 6 | 6 | 0 | 5 | 5 | 0 | 27 | adult | 14 | menopausal | 0 | 0 |
| Elli | 5 | 4 | 1 | 4 | 3 | 1 | 12 | adult | 5 | parous | 0 | 0 |
| Madeleine | 5 | 0 | 5 | 4 | 0 | 4 | 6 | adult | 9 | parous | 1 | 1 |
| Nils | 5 | 2 | 3 | 3 | 2 | 1 | 17 | adult | 7 | parous | 0 | 0 |
| Sandra | 5 | 4 | 1 | 4 | 3 | 1 | 8 | adult | 5 | nursing | 1 | 1 |
| Elisabeth | 4 | 4 | 0 | 4 | 4 | 0 | 4 | adolescent | 13 | nulliparous | 1 | 1 |
| Herta | 4 | 4 | 0 | 1 | 1 | 0 | 7 | adult | 6 | parous | 0 | 0 |
| Kurt | 4 | 3 | 1 | 4 | 3 | 1 | 5 | adult | 2 | nulliparous | 1 | 1 |
| Magdalena | 4 | 4 | 0 | 3 | 3 | 0 | 10 | adult | 4 | parous | 1 | 1 |
| Sabine | 4 | 4 | 0 | 4 | 4 | 0 | 14 | adult | 12 | parous | 0 | 0 |
| Bettina | 3 | 3 | 0 | 3 | 3 | 0 | 6 | adult | 10 | nursing | 0 | 0 |
| Maya | 3 | 3 | 0 | 3 | 3 | 0 | 12 | adult | 11 | nursing | 0 | 0 |
| Conchita | 2 | 2 | 0 | 2 | 2 | 0 | 5 | adult | 6 | nulliparous | 1 | 1 |
| Hermine | 2 | 1 | 1 | 2 | 1 | 1 | 3 | adolescent | 6 | nulliparous | 1 | 0 |
| Jessy | 2 | 2 | 0 | 2 | 2 | 0 | 9 | adult | 2 | nursing | 1 | 0 |
| Minni | 2 | 2 | 0 | 2 | 2 | 0 | 23 | adult | 4 | parous | 0 | 0 |
| Montana | 2 | 1 | 1 | 2 | 1 | 1 | 9 | adult | 2 | parous | 0 | 0 |
| Pia | 2 | 2 | 0 | 2 | 2 | 0 | 17 | adult | 2 | parous | 0 | 0 |
| Sakura | 2 | 2 | 0 | 2 | 2 | 0 | 6 | adult | 10 | parous | 1 | 1 |
| Zoey | 2 | 2 | 0 | 2 | 2 | 0 | 8 | adult | 7 | parous | 0 | 0 |
| Amanda | 1 | 1 | 0 | 1 | 1 | 0 | 26 | adult | 11 | menopausal | 0 | 0 |
| Julia | 1 | 0 | 1 | 1 | 0 | 1 | 7 | adult | 6 | nursing | 1 | 1 |
| Sailormoon | 1 | 1 | 0 | 1 | 1 | 0 | 3 | adolescent | 7 | nulliparous | 1 | 0 |
| Anastasia | 0 | 0 | 0 | 0 | 0 | 0 | 32 | adult | 9 | menopausal | 0 | 0 |
| Chita | 0 | 0 | 0 | 0 | 0 | 0 | 27 | adult | 4 | menopausal | 0 | 0 |
| Elektra | 0 | 0 | 0 | 0 | 0 | 0 | 3 | adolescent | 6 | nulliparous | 1 | 0 |
| Lucy | 0 | 0 | 0 | 0 | 0 | 0 | 6 | adult | 7 | nursing | 1 | 0 |
| Marylin | 0 | 0 | 0 | 0 | 0 | 0 | 5 | adult | 2 | nursing | 1 | 0 |
